# Role of omega-3 fatty acids in the prevention and treatment of cardiovascular Diseases: A consensus statement from the Experts’ Committee Of National Society Of Cardiometabolic Medicine

**DOI:** 10.3389/fphar.2022.1069992

**Published:** 2022-12-12

**Authors:** Jian-Jun Li, Ke-Fei Dou, Zhi-Guang Zhou, Dong Zhao, Ping Ye, Jia-Jun Zhao, Li-Xin Guo

**Affiliations:** ^1^ Cardiometabolic Center, Fuwai Hospital, Chinese Academy of Medical Sciences, Beijing, China; ^2^ Department of Metabolism and Endocrinology, The Second Xiangya Hospital of Central South University, Changsha, Hunan, China; ^3^ Department of Epidemiology, Beijing Anzhen Hospital Affiliated to Capital Medical University, Beijing, China; ^4^ Department of Cardiology of the First Medical Center, Chinese PLA General Hospital, Beijing, China; ^5^ Endocrine Department, Shandong Provincial Hospital, Jinan, Shandong, China; ^6^ Endocrine Department, Beijing Hospital, Beijing, China

**Keywords:** omega-3 fatty acids, cardiovascular disease, triglycerides, consensus, pharmacology

## Abstract

Low-density lipoprotein cholesterol (LDL-C) has been considered as the primary target for the prevention and treatment of atherosclerotic cardiovascular disease (ASCVD). However, there are still residual cardiovascular risks in some patients even if LDL-C achieves the target level. Emerging evidence suggestes that elevated triglyceride (TG) level or triglyceride-rich lipoprotein (TRL) cholesterol (TRL-C) is one of the important components of the residual cardiovascular risks. Omega-3 fatty acids have been shown to be one of the effective drugs for reducing TG. However, its efficacy in reducing the risk of ASCVD is inconsistent in large randomized clinical trials. There is lack of consensus among Experts regarding the application of omega-3 fatty acids in cardiovascular diseases including heart failure, arrhythmia, cardiomyopathy, hypertension, and sudden death. Hence, the current consensus will comprehensively and scientifically present the detailed knowledge about the omega-3 fatty acids from a variety of aspects to provide a reference for its management of omega-3 fatty acids application in the Chinese population.

## Introduction

Cardiovascular disease (CVD) is the leading cause of death and disability. Highest number of CVD deaths are reported in China annually, far exceeding those in India, Russia and United States (Roth et al., 2020). According to China Cardiovascular Health and Disease Report 2021, there are approximately 330 million patients suffering from CVD in China and CVD accounts for more than 40% of deaths in urban and rural China (The Writing Committee of the Report on Cardiovascular Health and Diseases in China, 2022). There is an immediate need for strengthening the measures of prevention and treatment.

Epidemiological, genomic, and population-based interventions have shown that elevated low-density lipoprotein cholesterol (LDL-C) is one of the major risk factors, a “pathogenicity”, for atherosclerotic cardiovascular disease (ASCVD). Current evidence suggests that lowering serum LDL-C levels, regardless of the method used, results in cardiovascular benefits. Therefore, LDL-C is the primary intervention target for ASCVD prevention and control. The introduction of statins to inhibit cholesterol synthesis, ezetimibe to reduce cholesterol absorption, and proprotein convertase subtilisin/kexin type 9 (PCSK9) inhibitors to increase cholesterol clearance has led to an unprecedented improvement in the ability to manage LDL-C and a major advance in the prevention and treatment of ASCVD (Joint Committee for Revising Chinese guidelines on Prevention and Treatment of Dyslipidemia in Adults, 2016). However, the observations presented through various randomized controlled trials show that an ideal level of LDL-C post statin therapy, in combination with ezetimibe and/or PCSK9 inhibitors has only resulted in 1/3rd reduction of cardiovascular events. The remaining 2/3rd cases which still occur after treating LDL-C are called residual cardiovascular (CV) risk (Cholesterol Treatment Trialists’ (CTT) Collaboration et al., 2010; Zambon, 2011; Dhindsa et al., 2020).

Although the cause of residual CV risk has not been fully understood, it is currently attributed to non-lipid and lipid related factors (Li, 2020). Non-lipid factors include hypertension, diabetes, inflammation, *etc.* Lipid factors primarily depend on whether LDL-C reaches the target level. Other lipid related targets include triglyceride (TG) lipoprotein (TRL), cholesterol (TRL-C) and lipoprotein 1) (Dhindsa et al., 2020; Li, 2020; Zhao, 2021; Li, 2022). Elevated TG or TRL-C is an important factor contributing to the increased residual cardiovascular risk, as per emerging evidence (Prevention Group of Cardiovascular Disease Branch of Chinese Medical Association, 2019). It is also one of the most common dyslipidemia phenotypes in Chinese population (Opoku et al., 2019).

Currently, there are relatively limited methods to significantly reduce TG in clinical practice, especially drug therapy. Omega-3 fatty acids have attracted attention in recent years. However, the results of recent large-scale clinical randomized controlled trials on the reduction of CVD risk by omega-3 fatty acids are not consistent. Consequently, the recommendations of the latest Chinese and global guidelines and/or consensus on lipids are not consistent, resulting in confusion in clinical practice in China. Hence, this Experts’ group conducted a review of the existing worldwide data regarding efficacy of omega-3 fatty acids and propose recommendations for use of omega-3 in Chinese clinical practice.

## Basic concepts of triglyceride and TRL-C

Blood lipid is a general term for lipids in human blood and refers to cholesterol, TG, phospholipids. The latter is combined with apolipoprotein and transported in plasma in the form of lipoprotein particles. Cholesterol is essential for cell structure and also for hormone synthesis while TG is used for energy storage and transportation. Elevated cholesterol and TG are related to the occurrence and development of ASCVD. Lipoprotein particles, in descending order of size, are chylomicrons (CM), very low-density lipoproteins (VLDL), intermediate density lipoproteins (ILDL), low density lipoprotein (LDL) and high-density lipoprotein (HDL) (Joint Committee for Revising Chinese guidelines on Prevention and Treatment of Dyslipidemia in Adults, 2016). The composition of cholesterol and TG in the various lipoprotein particles is different. The main component of large lipoprotein particles such as VLDL, ILDL and CM is TG, which is also called TRL. Under the action of enzymes related to lipid metabolism, the content of TG in TRL decreases and further forms small particles, which are called TRL remnants. The cholesterol contained in the remnant is called TRL-C (Ginsberg et al., 2021). TRL-C can be measured directly or calculated by the formula TRL-C = TC-high density lipoprotein cholesterol (HDL-C) -LDL-C (Wilson et al., 2021).

## Mechanisms and evidence of elevated triglyceride and TRL-C and increased residual risk of atherosclerotic cardiovascular disease

TG is a major biomarker of blood lipid and elevated TG is indicative of elevated TRL-C. According to recent Mendelian randomization studies and prospective longitudinal observations, after the therapeutic normalization of LDL-C, it was shown that TRL-C levels were causally associated with elevated CVD risk and also considered to be risk factor for residual CV risk. This contrasts with previous understanding that cholesterol, as a component of larger lipoprotein particles had little influence on the progression of ASCVD (Varbo et al., 2013). In the Women’s Health Study, TRL-C was causally associated with the development of ASCVD. The study also showed that the risk of ischemic stroke, peripheral arterial disease, and myocardial infarction increased by 30%, 160%, and 210%, respectively, in the largest quartile of TRL-C [≥ 1.6 mmol/L (61.0 mg/dl)] (Duran et al., 2020). The Experts’ consensus from the European Atherosclerosis Society (EAS) states that TRL-C varies with plasma TG levels, and that effective lipolysis limits remnant accumulation when TG is at an optimal level, i.e., TG < 1.2 mmol/L (100 mg/dl). When TG > 1.2 mmol/L (100 mg/dl), TRL-C begins to accumulate in the blood and enter the artery wall by a process called transcytosis. With elevated plasma levels, rate of influx exceed egress, resulting in accumulation of TRL-C in the subendothelial space. The subsequent degradation process may release bioactive lipids, causing endothelial dysfunction and inflammation. Parallelly, lipids are taken up by mononuclear macrophages to form foam cells, which result in formation of early atherosclerotic lesions. With repeated cycles of this process, lipid streaks form and gradually fuse into plaques, which when eroded or ruptured can lead to thrombosis and occlusion of the vascular lumen, leading to cardiovascular events (Ginsberg et al., 2021).

Thehe ACCORD study showed that in patients with normal post-statin treatment LDL-C levels, those who had TG ≥ 2.3 mmol/L (200 mg/dl) and low HDL-C levels had significantly higher cardiovascular risk than others (ACCORD Study Group et al., 2010). A series of studies from Chinese population confirmed that TRL-C levels were associated with cardiovascular events in patients with ASCVD (Zeng et al., 2017; Cao et al., 2020a; Cao et al., 2020b; Liu et al., 2021). In a prospective study conducted in 1,182 patients with type 2 diabetes undergoing elective percutaneous coronary intervention (PCI) to evaluate the association of TRL-C with myocardial injury after PCI, no association between TRL-C and myocardial injury after PCI was observed in group A [glycosylated hemoglobin (HbA_1c_) < 7%], but in group B [HbA_1c_≥ 7%], the risk of troponin I > 3 × upper limit of normal and >5 × upper limit of normal increased by 30% and 32%, respectively, for each standard deviation increase in TRL-C. It is suggested that TRL-C is an independent predictor of perioperative myocardial injury in patients with coronary heart disease undergoing PCI with poor glycemic control (Zeng et al., 2017). Another study showed that high levels of TRL-C are independent predictors of major adverse cardiovascular events in patients with coronary heart disease with diabetes and pre-diabetes. The study was conducted in 4,331 patients who were either diabetic, pre-diabetic or non-diabetic and suffered from coronary heart disease and were followed up for 5.1 years (Cao et al., 2020a). A Chinese real-world study, conducted in 4,355 patients with coronary heart disease and a median follow-up of 5.1 years, showed that the risk of cardiovascular events increased by 49%, 21% and 40% when the levels of TRL-C, remnant cholesterol and apolipoprotein C-III increased. The study also showed that elevated levels of TRL-associated biomarkers were independent predictors of cardiovascular event risk even when LDL-C was ≤1.8 mmol/L (70 mg/dl) (Cao et al., 2020b). In a recent population-based study in China, 6,732 statin-treated patients with coronary heart disease, TG < 2.3 mmol/L (200 mg/dl) and cardiovascular events were followed up for 59 months to evaluate the association between TRL-C and recurrent cardiovascular events, and whether inflammation affects the association. The results showed that high levels of TRL-C significantly increased the risk of recurrent cardiovascular events regardless of the level of high-sensitivity C-reactive protein (hs-CRP), suggesting that the role of TRL-C in predicting recurrent cardiovascular events in patients with coronary heart disease is independent of inflammatory status (Liu et al., 2021).

In particular, the 2021 EAS consensus statement on TRL-C emphasizes that a large number of epidemiological and genetic studies have shown that elevated TG and TRL-C levels are causally associated with cardiovascular disease, and that elevated TG is an important component of the residual risk of ASCVD (Ginsberg et al., 2021). A large number of previous studies have also confirmed that omega-3 fatty acids can significantly reduce TG levels. However, data from randomized controlled trials studying the association between omega-3 and it’s impact on cardiovascular events is contradictory. This contradiction has become the basis of academic debate and is the purpose of writing this Experts’ consensus. This multitude of scientific evidence is summarized in Table 1. Experts’ opinion 1: Elevated TG, and TRL-C, levels are independently and positively associated with the risk of cardiovascular disease and major events (causal association), which may be one of the reasons for the residual risk of ASCVD.

**TABLE 1:** Mechanisms and evidence of elevated TG and TRL-C in reference to increased residual risk of ASCVD.

Study (s)	Outcomes	Author
Mendelian randomization studies and prospective longitudinal observations of the population	Elevated TRL-C levels are causally associated with increased cardiovascular risk and are considered to be a lipid risk factor for residual cardiovascular risks after LDL-C levels are ideally regulated	Varbo et al. (2013)
Women’s Health Study	TRL-C was causally associated with the development of ASCVD, and the risk of ischemic stroke, peripheral arterial disease, and myocardial infarction increased by 30%, 160%, and 210%, respectively, in the largest quartile of TRL-C [≥ 1.6 mmol/L (61.0 mg/dl)]	Duran et al. (2020)
The expert consensus from the European Atherosclerosis Society (EAS)	When TG > 1.2 mmol/L (100 mg/dl), TRL-C begins to accumulate in the blood and enters the artery wall by transcytosis, starting a process which may lead to cardiovascular events such as acute coronary syndrome	Ginsberg et al. (2021)
ACCORD study	Patients with LDL-C targets after statin therapy, those with TG ≥ 2.3 mmol/L (200 mg/dl) and low HDL-C levels had significantly higher cardiovascular risk	ACCORD Study Group et al. (2010)
Chinese population-based studies	TRL-C levels were associated with cardiovascular events in patients with ASCVD.	Zeng et al. (2017); Cao et al. (2020a); Cao et al. (2020b); Liu et al. (2021)
A prospective study of 1,182 patients with type 2 diabetes undergoing elective percutaneous coronary intervention (PCI)	It is suggested that TRL-C is an independent predictor of perioperative myocardial injury in patients with coronary heart disease undergoing PCI with poor glycemic control	Zeng et al. (2017)
A prospective study of 4,331 patients with different glucose metabolic status and with coronary heart disease followed up for an average of 5.1 years	It was suggested that high levels of TRL-C are independent predictors of major adverse cardiovascular events in patients with coronary heart disease with diabetes and pre-diabetes	Cao et al. (2020a)
A real-world Chinese population-based study included 4,355 patients with coronary heart disease treated with statins, with a median follow-up of 5.1 years	The results showed that the risk of cardiovascular events increased by 49%, 21% and 40% when the levels of TRL-C, remnant cholesterol and apolipoprotein C-III increased. In addition, elevated levels of TRL-associated biomarkers were independent predictors of cardiovascular event risk even when LDL-C was ≤1.8 mmol/L (70 mg/dl)	Cao et al. (2020b)
A population-based study in China, 6,732 statin-treated patients with coronary heart disease TG < 2.3 mmol/L (200 mg/dl) and cardiovascular events with a follow-up of 59 months	The results showed that high levels of TRL-C significantly increased the risk of recurrent cardiovascular events regardless of the level of high-sensitivity C-reactive protein (hs-CRP), suggesting that the role of TRL-C in predicting recurrent cardiovascular events in patients with coronary heart disease is independent of inflammatory status	Liu et al. (2021)
The 2021 EAS consensus statement	A large number of epidemiological and genetic studies have shown that elevated TG and TRL-C levels are causally associated with cardiovascular disease, and that elevated TG is an important component of the residual risk of ASCVD.	Ginsberg et al. (2021)

## Omega-3 fatty acids overview

Omega-3 fatty acids are essential polyunsaturated fatty acids, named for their first double bond at the third carbon atom at the end of the methyl group (Saini and Keum, 2018). Omega-3 fatty acids mainly include alpha-linolenic acid, eicosapentaenoic acid (EPA), and docosahexaenoic acid (DHA). Alpha-linolenic acid is derived from plants, while EPA and DHA are mainly derived from fish, krill and squid in the ocean (Barry and Dixon, 2021). Omega-3 fatty acid preparations are currently divided into over-the-counter fish oil products and prescription omega-3 fatty acid products according to their composition, purity and dosage differences. Over-the-counter fish oil products are classified as dietary supplements and cannot be substituted for prescription omega-3 fatty acid products, as over the counter and prescription products have different regulations, evaluation, composition and purity, efficacy and safety (Preston Mason, 2019; Virani et al., 2021).

There are currently three prescription omega-3 fatty acid products on the market: 1) omega-3 fatty acid ethyl ester, the main components of which are EPA and DHA; 2) icosapent ethyl (IPE), the main component of which is the ethyl ester of EPA; 3) omega-3 carboxylic acids, a mixture of long-chain omega-3 fatty acids in the form of free fatty acids, the principal components being EPA, DHA, and docosapentaenoic acid (Backes et al., 2016).

## Evidence for 4 omega-3 fatty acids in cardiovascular disease prevention

### Atherosclerotic cardiovascular disease

The clinical value of omega-3 fatty acids in the prevention and treatment of ASCVD has been explored for a long time and highlighted in Table 2. More than 30 years ago, the DART study found that regular intake of deep-sea fatty fish or fish oil capsules in patients with recent myocardial infarction reduced all-cause mortality rate by 29% (Burr et al., 1989). The GISSI-P study, the first large randomized controlled study of omega-3 fatty acids, showed that omega-3 fatty acids (EPA/DHA = 1:2) significantly reduced the risk of the primary composite endpoint of all cause death, nonfatal myocardial infarction, and nonfatal stroke by 15% in patients with recent myocardial infarction (GISSI-Prevenzione Investigators, 1999). The JELIS study in 2007 included 18,645 Japanese patients aged 40–75 years who were on statin therapy and had total cholesterol ≥6.5 mmol/L (251 mg/dl) and were randomized to 1.8 g/d of EPA ethyl ester plus statin or statin alone, with a mean follow-up of 4.6 years. The results showed that major coronary events [sudden cardiac death, fatal or nonfatal myocardial infarction, unstable angina (UA), and coronary revascularization] were significantly reduced in the EPA ethyl ester group by 19% (2.8% vs. 3.5%, HR = 0.81, 95% CI: 0.69–0.95) (Yokoyama et al., 2007).

**TABLE 2:** Omega-3 fatty acids for prevention and treatment of atherosclerotic cardiovascular diseases.

Study/Year	Region	Subject	Background treatment	Study group	Control group	Follow-up	Primary endpoint and results
GISSI-P/1999	Italy	Patients with myocardial infarction within 3 months (n = 11,324)	Fish (≥1 serving/week): 87.6%	EPA + DHA	No interven-tion	3.5 years	The primary combined efficacy endpoint was death, non-fatal myocardial infarction and stroke. There was a 15% risk reduction in the primary endpoint; Benefit was attributable to a 20% risk reduction of deathand 30% reduction in cardiovascular death
			Cholesterol-lowering drugs: 45.5%	1 g/d			
			Antihypertensive or antiplatelet agents: 38.5%–82.8%				
			Revascularization: 24%				
JELIS/2007	—	Patients with total cholesterol ≥6.5 mmol/L (251 mg/dl) (*n* = 18,645)	Statins: 100%	EPA	No intervention	4.6 years	Primary endpoints: major adverse coronary events (MACE), defined as sudden cardiac death, unstable angina, myocardial infarction, or revascularization. Primary endpoint of MACE was significantly lower in EPA plus statin vs. statin alone (2.8% vs. 3.5% *p* = 0.011) Among the components of the composite endpoint, the EPA plus statin group had lower rates of unstable angina (1.6% vs. 2.1%, HR 0.76, *p* = 0.014) and a trend toward lower rates of nonfatal MI (0.7% vs. 0.9%, *p* = 0.086) and revascularization (2.1% vs. 2.4%, *p* = 0.135) with no difference in sudden cardiac death (0.2% each) or fatal MI (0.1% vs. 0.2%, *p* = NS)
			Antihypertensive or antiplatelet agents: ≤30%	1.8 g/d			
			Revascularization: 5%				
ASCEND/2018	The United Kingdom	Diabetic patients without atherosclerotic cardiovascular disease (*n* = 15,480)	Statins: about 75%	EPA + DHA	Olive oil	7.4 years	The primary efficacy outcome, major adverse cardiovascular events (vascular death, myocardial infarction, or stroke/transient ischemic attack), occurred in 8.9% of the omega-3 group compared with 9.2% of the placebo group (*p* = 0.55).Omega-3 fatty acid failed to reduce any severe vascular events *versus* placebo
			Antiplatelet agents: about 36%	1 g/d			
VITAL/2019	America	Men (≥50 years) or women (≥55 years) without cardiovascular disease and cancer (*n* = 25,871)	Fish (≥1.5 servings/week): 47%	EPA + DHA	Placebo (composition unknown)	5.3 years	There was no reduction in the co-primary endpoint of major CVD events [a composite of MI, stroke, and CVD mortality; HR = 0.97 (0.85–1.12)] [2]. It also did not reduce prespecified secondary cardiovascular endpoints, including an expanded composite of major CVD events plus coronary revascularization (HR = 0.96 [0.86–1.08]), or myocardial infarction (HR = 0.96 (0.78–1.19)], stroke [HR = 0.95 (0.76–1.20)], and CVD mortality (HR = 1.11 [0.88–1.40]) considered individually. Also there was no effect on all-cause mortality [HR = 0.99 (0.87–1.12)]
			Cholesterol-lowering drugs: 37.5%	1 g/d			
			Antihypertensive or antiplatelet agents: about 45%–50%				
REDUCE-IT/2019	International	Patients with cardiovascular disease or type 2 diabetes with cardiovascular risk factors and fasting triglycerides of 1.5–5.6 mmol/L (135–499 mg/dl) and LDL-C of 1.1–2.6 mmol/L (41–100 mg/dl) after statin therapy (*n* = 8,179)	Statins: >99%	IPE	Mineral oil	4.9 years	The primary endpoint of 5-point MACE (cardiovascular death, MI, stroke, coronary revascularization, or hospitalization for unstable angina) was reduced from 22.0% to 17.2% [hazard ratio (HR) 0.75, 95% confidence interval (CI) 0.68–0.83; *p* = 0.00000001]. This translates into a number needed to treat of only 21. The key secondary endpoint of cardiovascular death, MI, or stroke was reduced from 14.8% to 11.2% (HR 0.74, 95% CI 0.65–0.83; *p* = 0.0000006), which translates to a number needed to treat of only 28
				4 g/d			
STRENGTH/2020	International	Statin-treated patients at high cardiovascular risk with hypertriglyceridemia and low HDL-C (*n* = 13,078)	Statins: 100%	Omega-3 carboxylic acid 4 g/d	Corn oil	3.5 years	The primary efficacy measure was a composite of cardiovascular death, nonfatal myocardial infarction, nonfatal stroke, coronary revascularization, or unstable angina requiring hospitalization Trial was prematurely halted based on an interim analysis that indicated a low probability of clinical benefit of Omega-3 CA vs. the cornoil comparator. The primary end point occurred in 785 patients (12.0%) treated with omega-3 CA vs. 795 (12.2%) treated with corn oil (hazard ratio, 0.99 [95% CI, 0.90–1.09]; *p* = 0.84)
			Antihypertensive or antiplatelet agents: >70%				
OMEMI/2021	Norway	Patients aged 70–82 years with recent acute myocardial infarction (2–8 weeks) (*n* = 1,027)	Statins: about 96%	EPA + DHA	Corn oil	2 years	The primary outcome of all-cause death, nonfatal myocardial infarction, unscheduled revascularization (stent or bypass surgery), stroke, or hospitalization for heart failure at 24 months occurred in 21.0% of the EPA + DHA group compared with 19.8% of the placebo group (*p* = 0.62). The treatment effect was the same in tested subgroups
			Antihypertensive or antiplatelet agents: >80%	1.8 g/d			

Note: EPA, eicosapentaenoic acid; DHA, docosahexaenoic acid; IPE, icosapent ethyl; LDL-C, low-density lipoprotein cholesterol; HDL-C, High-density lipoprotein cholesterol.

However, several subsequent randomized controlled studies of omega-3 fatty acids have not yielded positive results (Rauch et al., 2010; ORIGIN Trial Investigators et al., 2012; Risk and Prevention Study Collaborative Group et al., 2013). The ASCEND study showed that omega-3 fatty acids (460 mg EPA + 380 mg DHA) did not significantly reduce the risk of serious vascular events [nonfatal myocardial infarction or stroke, transient ischemic attack (TIA), or vascular death] (8.9% vs. 9.2%, RR = 0.97,95%CI: 0.97–1.08). The study was published in 2018 and was a randomized, placebo-controlled trial that included 15,480 diabetic patients aged ≥40 years without CVD in the United Kingdom, with an average follow-up of 7.4 years. (ASCEND Study Collaborative Group et al., 2018). The VITAL study, published in 2019, showed that omega-3 fatty acids (460 mg EPA + 380 mg DHA) did not significantly reduce the risk of major cardiovascular events (myocardial infarction, stroke, or cardiovascular death) (3.0% vs. 3.2%, HR = 0.92, 95% CI: 0.80–1.06). The trial was a randomized, placebo-controlled, 2 × 2 factorial design trial that included 25,871 patients without known CVD or cancer in the United States with a mean follow-up of 5.3 years (Manson et al., 2019). The findings raised questions about the value of omega-3 fatty acids in patients with ASCVD. However, the REDUCE-IT study, published in 2019, showed good cardiovascular benefits. Since then, the use of omega-3 fatty acids in CVD was once again attracting attention. REDUCE-IT was a multicenter, randomized, double-blind, placebo-controlled trial of IPE (4 g/d) or placebo (mineral oil) in 8,179 patients with CVD or type 2 diabetes + ≥ 1 cardiovascular risk factor on a clinical setting of elevated TG levels despite statin therapy. The median follow-up was 4.9 years. The results showed that IPE significantly reduced the risk of the primary endpoint (the composite of cardiovascular death, nonfatal myocardial infarction, nonfatal stroke, coronary revascularization, or UA) by 25% (17.2% vs. 22%, HR = 0.75, 95% CI: 0.68–0.83), and also reduced cardiovascular death (4.3% vs. 5.2%, HR = 0.80, 95% CI: 0.66–0.98), fatal or nonfatal myocardial infarction (6.1% vs. 8.7%, HR = 0.69, 95% CI: 0.58–0.81) and fatal or nonfatal stroke (2.4% vs. 3.3%, HR = 0.72, 95% CI: 0.55–0.93), but there was no reduction in the risk of all-cause mortality (Bhatt et al., 2019).

Paradoxically, the STRENGTH and OMEMI studies published after the REDUCE-IT study again showed negative results for cardiovascular effects. STRENGTH was a multicenter, double-blind, placebo-controlled, randomized clinical trial in 22 countries involving 13,078 patients at high cardiovascular risk with hypertriglyceridemia (HTG) and low HDL-C, who were randomized to omega-3 fatty acids (omega-3 carboxylic acids) or placebo (corn oil). After a median follow-up of 3.5 years, the study was terminated early after the mid-term analysis showed no statistically significant difference between the two groups in major adverse cardiovascular events ((MACE) cardiovascular death, nonfatal myocardial infarction, nonfatal stroke, coronary revascularization or hospitalization for UA) (12% vs. 12.2%, HR = 0.99, 95% CI: 0.90–1.09) (Nicholls et al., 2020). The OMEMI study was a multicenter, randomized, double-blind, placebo-controlled clinical trial in 1,027 elderly Norwegian patients with recent acute myocardial infarction who were followed up for 2 years. The results showed that omega-3 fatty acids (930 mg EPA +660 mg DHA) did not reduce the risk of the primary endpoint (nonfatal acute myocardial infarction, unplanned revascularization, stroke, all-cause death, or hospitalization for heart failure) (21.4% vs. 20%, HR = 1.07, 95% CI: 0.82–1.40) (Kalstad et al., 2021).

Thus, early studies have shown that omega-3 fatty acids have cardiovascular benefits, and the subsequent negative results of a number of randomized controlled studies have raised concern whether omega-3 fatty acids can be beneficial for prevention of CVD. Although the reason is unknown, some scholars speculate that the proportion of fish intake and optimized cardiovascular treatment (such as statins, antihypertensive or antiplatelet agents, revascularization) in the past decade is much higher than that in the early stage, and good treatment may weaken the cardiovascular benefits of omega-3 fatty acids (Siscovick et al., 2017).

It is worth pointing out that the REDUCE-IT study, 12 years later, once again confirmed that omega-3 fatty acids can reduce the risk of major cardiovascular events in high-risk groups, which has aroused extensive and in-depth discussion on the results of different studies. One of the major points of discussion is over the different components of omega-3 fatty acids in trials and studies. A recent meta-analysis showed that EPA alone can be more effective at reducing CVD risk, compared to EPA + DHA.

Khan et al. (2021) According to the mechanism of EPA and DHA, the level of serum EPA may be the key to the clinical benefit of IPE. In the JELIS and REDUCE-IT studies, serum EPA levels were inversely associated with cardiovascular risk in a dose-response relationship. In addition, further analysis of the REDUCE-IT study showed that most of the relative risk reduction observed in the IPE group came from changes in EPA concentrations, not lipid biomarkers (Itakura et al., 2011; Bhatt et al., 2020). EPA levels in the STRENGTH study were much lower than those in the REDUCE-IT and JELIS studies (89.6 μg/ml vs. 135.2 μg/ml vs. 170 μg/ml) (Nissen et al., 2021). Evidence suggests that serum EPA levels may need to reach a certain threshold for it to be efficacious, hence omega-3 fatty acids with a higher content of EPA may be required. Meta-analyses have shown that the effect of omega-3 fatty acids on reducing the risk of events increases with increasing dose (Abdelhamid et al., 2020). Higher doses of EPA (>1 g/d) significantly reduced the risk of cardiovascular events compared with lower doses (≤1 g/d) (Lombardi et al., 2020). However, secondary analysis of the STRENGTH study showed that EPA did not affect cardiovascular risk at the highest tertile level, which does not seem to support this hypothesis. It is worth noting that the DHA level of the highest EPA group were also high, and the increase in DHA levels may reduce part of the protective effect of EPA (Bhatt et al., 2020). Similar outcomes were reported in the INSPIRE study, which showed that higher DHA levels impaired the cardiovascular benefits of EPA when administered as a combination (Viet, 2021). This is one explanation for the lack of cardiovascular benefit in the STRENGTH study.

With regard to the existing doubts about the use of mineral oil as a control group in the REDUCE-IT study, the biomarker subgroup data from the REDUCE-IT study recently published in *Circulation* showed that by 12 months, compared with baseline levels, the levels of homocysteine, lipoprotein (a), oxidized LDL-C (Ox-LDL-C), interleukin-6 (IL-6), lipoprotein-associated phospholipase A2 (Lp-PLA2), hs-CRP and interleukin-1β (IL-1β) in the mineral oil group increased by 1.5%, 2.2%, 10.9%, 16.2%, 18.5%, 21.9%, and 28.9% respectively, and the changes of various biomarkers were similar at 24 months of follow-up. In the pure fish oil preparation group, there were no significant changes in the levels of these biomarkers at either 12 or 24 months of follow-up. The study concluded that the implications of these new findings for explaining the reduction in the total risk of clinical events observed in the REDUCE-IT study are uncertain (Ridker et al., 2022). However, it is suggested that the mineral oil as the control group in the REDUCE-IT study may have insufficient scientific consideration, or that the mineral oil used as the placebo may have a negative impact on the cardiovascular risk of the control group by increasing atherosclerotic lipids (such as apolipoprotein B, LDL-C) and hs-CRP, or reducing the absorption of statins (Sharma et al., 2020; Barry and Dixon, 2021; Iqbal and Miller, 2021). In addition, (Doi et al., 2021), reviewed the clinical data of 106,888 cases in Copenhagen and conducted a simulation experiment on the REDUCE-IT study. The results showed that mineral oil was associated with a 7% increased risk of ASCVD after adjustment for TG, LDL-C, and hs-CRP. However, the United States Food and Drug Administration (FDA) Endocrinologic and Metabolic Drugs Advisory Committee, in its review of the IPE, concluded that changes in LDL-C and hs-CRP in the placebo group had little effect on the achievement of the primary endpoint and were unlikely to alter the overall results of the study (Meeting of the Endocrinologic and Metabolic Drugs Advisory Committee meeting announcement, 2019). Some scholars have also conducted retrospective analysis that mineral oil does not seem to affect drug absorption or efficacy or related clinical outcomes. Therefore, the use of a certain amount of mineral oil as placebo in clinical studies may have a relatively limited impact on the conclusion of the study (Olshansky et al., 2020). At the same time, recent studies showed no significant difference in coronary plaque volume progression, an intermediate endpoint, between the mineral oil and non-mineral oil placebo groups (Lakshmanan et al., 2020). In addition, significant improvements in plaque and CVD events were still observed with highly purified EPA in studies that did not use mineral oil, such as CHERRY and JELIS (Yokoyama et al., 2007; Watanabe et al., 2017). In fact, it has also been suggested that the corn oil placebo used in the STRENGTH and OMEMI study may have been beneficial because it was shown to reduce atherogenic lipoprotein particles compared to extra virgin olive oil (Maki et al., 2015; Maki et al., 2017). However, corn oil was not commonly used as a placebo in all EPA + DHA studies with negative results. Therefore, in the light of existing observations, the likelihood of differences in the results of large randomized controlled studies of omega-3 fatty acids due to different placebo selections is relatively small (Iqbal and Miller, 2021). However, the cardiovascular benefits of omega-3 fatty acids may need to be further consolidated in a new randomized controlled study, as noted in a commentary published by the *Circulation* Editorial Board on a subgroup of blood markers from the REDUCE-it study (Harrington, 2022).

Experts’ opinion 2: Factors such as the purity and content of EPA in omega-3 fatty acids and blood EPA levels in treated subjects may be the main reasons for the difference in cardiovascular risk and benefit of omega-3 fatty acids in different randomized controlled studies. The effect of different placebos (corn oil or mineral oil) in the control group needs further study.

### Heart failure

Studies suggest that omega-3 fatty acids may be beneficial in the prevention and treatment of heart failure. The GISSI-HF study, a previous large randomized controlled study, showed that omega-3 fatty acids significantly reduced the risk of all-cause mortality by 9% (*p* = 0.041) and the rate of all-cause mortality or cardiovascular hospitalization by 8% (*p* = 0.009) over 3.9 years (Tavazzi et al., 2008). Based on the GISSI-HF study, the American Heart Association (AHA) in a scientific statement in 2017 on omega-3 fatty acids for the prevention of clinical CVD recommended that omega-3 fatty acids are reasonable for secondary prevention in patients with heart failure (IIa, B) (Siscovick et al., 2017). The 2019 MESA cohort study found a negative correlation between plasma EPA abundance (percentage of EPA) and the occurrence of heart failure. The study also established a model for predicting the occurrence of heart failure with plasma EPA abundance. The study included 6,562 participants with a total of 292 heart failure events during a median follow-up of 13 years, with plasma EPA abundance of 0.76% and 0.69% for those without and with heart failure, respectively (*p* = 0.005) (Block et al., 2019). The newly published *post hoc* analysis of the VITAL-HF study showed that omega-3 fatty acids significantly reduced the risk of first and recurrent heart failure hospitalization by 31% and 47%, respectively, in patients with diabetes mellitus (Djoussé et al., 2022). A recent meta-analysis of 12 randomized controlled studies involving 81,364 patientsshowed that omega-3 fatty acids did not significantly reduce the risk of first heart failure hospitalization and cardiovascular death, but significantly reduced the risk of recurrent heart failure hospitalization by 9% (*p* = 0.01) (Barbarawi et al., 2021). The 2022 AHA/American College of Cardiology (ACC)/Heart Failure Society of America (HFSA) Guideline for the Management of Heart Failure recommends omega-3 fatty acids for patients with heart failure (NYHA Class Ⅱ-Ⅳ) to reduce the risk of cardiovascular hospitalization and mortality (IIb, B), which is the first time that omega-3 fatty acids have been recommended from the perspective of heart failure treatment (Heidenreich et al., 2022).

### Arrhythmia

Results of previous randomized controlled trials do not support that omega-3 fatty acids reduce the risk of recurrent atrial fibrillation (AF) in patients with AF (Kowey et al., 2010; Macchia et al., 2013; Nigam et al., 2014; Darghosian et al., 2015). There is also no support for reducing the risk of AF after cardiothoracic surgery (Heidarsdottir et al., 2010; Saravanan et al., 2010; Mozaffarian et al., 2012; Sandesara et al., 2012; Joss et al., 2017). In the recently released VITAL Rhythm study, no statistically significant difference in the incidence of AF between omega-3 fatty acids and placebo in people without prior CVD, cancer, or AF (3.7% vs. 3.4%, *p* = 0.19) was reported (Albert et al., 2021). Omega-3 fatty acids not only fail to reduce the risk of AF but may also be related to increased risk of AF. The latest meta-analysis showed that omega-3 fatty acids increased the relative risk of AF by 25% in randomized controlled studies assessing cardiovascular outcomes (Gencer et al., 2021).

There are contradictory findings on omega-3 fatty acids regarding morbidity or mortality associated with ventricular arrhythmias. Of the two randomized controlled studies published in 2005, one study showed that omega-3 fatty acids did not reduce the risk of ventricular tachycardia/fibrillation in patients with implantable cardioverter defibrillators (ICDs) and may even increase the risk of arrhythmias in some patients (Raitt et al., 2005). The other study showed that omega-3 fatty acids have benefits in ICD patients, especially in high-risk groups (Leaf et al., 2005). The SOFA study showed no benefit of omega-3 fatty acids on ventricular arrhythmias in patients with ICD (Brouwer et al., 2006). A randomized controlled study conducted in 2017 evaluated the effect of omega-3 fatty acids on ventricular tachyarrhythmia episodes (VTE) in patients with ICD and ischemic cardiomyopathy. The results showed that the average number of VTE in the omega-3 fatty acid group was significantly lower than that in the placebo group (1.7 vs. 5.6, *p* = 0.035) (Weisman et al., 2017).

### Cardiomyopathy

There is inadequate evidence of omega-3 fatty acids in patients with cardiomyopathy, especially in adults, and no large randomized controlled studies have been conducted. Existing evidence suggests that omega-3 fatty acids can improve cardiac function, such as left ventricular ejection fraction and left ventricular end-diastolic diameter, in children with dilated cardiomyopathy (Olgar et al., 2007; Firuzi et al., 2013). It can also improve the cardiac function of children with ventricular premature beats, thereby preventing reversible cardiomyopathy (Oner et al., 2018). In addition, omega-3 fatty acids reverse tachycardia-mediated atrial cardiomyopathy in adults (Kumar et al., 2011), and has a beneficial effect on inflammatory markers and lipid levels in adult patients with Chagas cardiomyopathy (Silva et al., 2017).

### Hypertension

High-dose dietary fish oil (containing 15 g of omega-3 fatty acids) is effective in lowering blood pressure in patients with mild hypertension, as compared to vegetable oils, according to previously conducted studies (Knapp and FitzGerald, 1989; Levinson et al., 1990). Several subsequent long-term cohort studies and short-term randomized controlled studies have confirmed that increased omega-3 fatty acid intake is associated with lower blood pressure in hypertensive patients, but the evidence is insufficient in normotensive people (Bercea et al., 2021). A meta-analysis of 70 randomized controlled studies showed that omega-3 fatty acids reduced blood pressure by 1.52/0.99 mmHg (1 mmHg = 0.133 kPa) compared with placebo. The decrease was 1.25/0.62 mmHg and 4.51/3.05 mmHg in normotensive and hypertensive patients, respectively (Miller et al., 2014). Another meta-analysis of 71 randomized controlled studies involving 4,973 patients that evaluated the relationship between omega-3 fatty acid dose and antihypertensive efficacy showed that the optimal dose of omega-3 fatty acids for lowering blood pressure ranged from 2 g/d (−2.61/−1.64 mmHg) to 3 g/d (−2.61/−1.80 mmHg), with a stronger and nearly linear dose-response relationship in the hypertensive, hyperlipidemic, and elderly population (Zhang et al., 2022).

### Sudden cardiac death

Earlier evidence showed that omega-3 fatty acids are associated with reduced risk of sudden cardiac death by 45%–81% (Carroll and Roth, 2002). However, a meta-analysis of 20 studies involving 68,680 patients concluded that omega-3 fatty acids did not reduce sudden cardiac death (RR = 0.91, 95% CI: 0.89–0.98) or sudden death (RR = 0.87, 95% CI: 0.75–1.01) (Rizos et al., 2012). Further analysis found that the benefit of omega-3 fatty acids on sudden cardiac death may be related to background therapy, circulating/tissue omega-3 fatty acid levels, and other factors. A meta-analysis of 10 randomized controlled studies involving 33,429 CVD patients evaluated the role of omega-3 fatty acids in sudden cardiac death. The results showed that omega-3 fatty acids did not reduce the risk of sudden cardiac death in patients receiving guideline-adjusted therapy, but significantly reduced the risk of sudden cardiac death by 36% in patients not receiving guideline-adjusted therapy (Chen et al., 2011). A case-cohort study of 203 patients with cardiovascular death after non-ST-segment elevation acute coronary syndrome, 325 patients with myocardial infarction, 271 patients with ventricular tachycardia, and 161 patients with AF from the MERLIN-TIMI 36 study was compared with a random sample of 1,612 event-free patients. After adjustment for all conventional risk factors, those with higher baseline plasma levels of long-chain omega-3 fatty acids (highest quartile) had 51% and 63% reductions in cardiovascular mortality and sudden cardiac death, respectively (Zelniker et al., 2021). Two other pooled analyses included 17 (median follow-up 16 years) and 19 (median follow-up 10 years) cohort studies, after multivariable adjustment for relevant risk factors. The results showed that those in the highest quintile of long chain omega-3 fatty acids had a 15%–21% lower risk of cardiovascular death than those in the lowest quintile (*p* < 0.01) (Harris et al., 2021). A standard deviation increase in circulating/tissue omega-3 fatty acid levels was associated with a 9%–10% reduction in the risk of fatal coronary heart disease (Del Gobbo et al., 2016).

Experts’ opinion 3: Current evidence only supports that high-purity and high-dose EPA therapy may bring cardiovascular benefits to ASCVD patients, and recent guidelines recommend omega-3 fatty acids for heart failure (NYHA Class II-IV) patients to reduce the risk of cardiovascular hospitalization and death (IIb, B). However, the role of omega-3 fatty acids in the treatment of other CVD such as arrhythmia, cardiomyopathy, hypertension, sudden cardiac death and so on needs further study.

## Cardiovascular protective mechanisms of omega-3 fatty acids

Although the cardiovascular benefits of omega-3 fatty acids are controversial in clinical application, the research into the mechanism of cardiovascular protection by omega-3 fatty acids is still rigorously being pursued.

### Lipid-lowering effect

The effect of omega-3 fatty acids in reducing the risk of ASCVD events is partially associated with a reduction in TG levels (Marston et al., 2019). Omega-3 fatty acids decrease serum TG levels by reducing TG synthesis, reducing TG incorporation into VLDL, reducing TG secretion, and enhancing TG clearance from VLDL particles (Backes et al., 2016). Omega-3 fatty acids (4 g/d) were shown to reduce TG levels by about 20%–30% and ≥30% in patients with TG of 2.3–5.6 mmol/L (200–499 mg/dl) and ≥5.6 mmol/L (500 mg/dl), respectively (Skulas-Ray et al., 2019). Moreover, the efficacy of different omega-3 fatty acid products in reducing TG is comparable (Kelley and Adkins, 2012; Backes et al., 2016). Omega-3 fatty acids reduce the production of VLDL during TG reduction, thereby reducing plasma TRL-C levels, thus reducing the probability of atherogenesis. Therefore, the existing scientific interpretation suggests that part of the benefit of REDUCE-IT research may come from the change of TRL-C level.

Non-high-density lipoprotein cholesterol (non-HDL-C) levels are also strongly associated with cardiovascular risk and are reduced in hypertriglyceridemic patients treated with omega-3 fatty acids (Marston et al., 2019) which may reflect an improvement in the risk of atherosclerosis. EPA + DHA studies showed a 5%–7% reduction in non-HDL-C while IPE studies showed a 12%–14% reduction in non-HDL-C (Skulas-Ray et al., 2019). It is worth noting that EPA + DHA has been reported to increase LDL-C levels by about 15%–36%. Since no change in apolipoprotein B levels was seen in the study results, it is speculated that the increase in LDL-C may reflect an increase in LDL particle diameter rather than an increase in LDL particle concentration or number (Kelley and Adkins, 2012; Skulas-Ray et al., 2019). In addition, the cardiovascular benefits of high-dose EPA seem to exceed the expected lipid-lowering effects in several clinical studies, suggesting that there may be cardiovascular protective mechanisms other than lipid-lowering effects (Marston et al., 2019).

### Non-lipid-lowering effect

In addition to regulating blood lipids, omega-3 fatty acids can also play an anti-atherosclerotic role by regulating endothelial function, membrane stability, inflammation and adhesion molecules, lipid peroxidation, reducing plaque formation and stabilizing plaque, reducing platelet activation and aggregation, and regulating blood pressure and heart rate (Kelley and Adkins, 2012; Sheikh et al., 2019; Zambon et al., 2020). Notably, EPA and DHA have different tissue distributions and affect target organs in different ways. EPA mainly acts on blood vessels, while DHA is abundant in nerve tissue and has a significant impact on neurons and retinal membrane tissue.

EPA has a lipophilic and stable extended conformation, which can reduce membrane fluidity, maintain stable membrane structure and uniform cholesterol distribution, regulate inflammation and endothelial dysfunction, and inhibit free radicals and lipid oxidation. The sustained antioxidant effects of EPA cannot be replicated by other TG lowering drugs When combined with statins, the effect is enhanced, which significantly inhibits the formation of membrane cholesterol crystalline domains, thus stabilizing plaques and playing a direct protective role in the progression of atherosclerosis (Preston Mason, 2019; Sheikh et al., 2019; Wang et al., 2020; Zambon et al., 2020; Mason and Eckel, 2021). The CHERRY study showed that statins combined with EPA significantly reduced coronary plaque volume and increased plaque stability (Watanabe et al., 2017). The EVAPORATE study confirmed that the combination of IPE and statins reduced the volume of coronary low-attenuation plaques (unstable plaques) by 17% (*p* = 0.0061) (Budoff et al., 2020).

DHA undergoes rapid conformational changes in the cell membrane, which is not conducive to the maintenance of cell membrane stability. Its anti-lipid oxidation activity is relatively limited. In addition, DHA cannot reduce the formation of cholesterol-rich domains, thereby inhibiting the formation of extracellular toxic crystals. Like oxidized LDL, cholesterol crystallization can also activate the active cytokines of atherosclerotic inflammation, thereby inducing apoptosis and necrosis and causing plaque instability (Kelley and Adkins, 2012; Preston Mason, 2019; Sheikh et al., 2019; Zambon et al., 2020; Mason and Eckel, 2021).

Studies have shown that another difference from DHA is that EPA is a 3-series prostaglandin [B_3_, D_3_, E_3_, I_3_, and thromboxane A_3_ (TXA_3_)] precursor, which is metabolized by cyclooxygenase-2 (COX-2) and 5-lipoxygenase (5-LOX). Endothelium-derived vasodilatory mediators [prostaglandin I_3_ (PGI_3_)] have potent vasodilatory effects, and can reduce platelet aggregation, reduce cardiac ischemic injury, reduce arteriosclerosis, and promote angiogenesis (Saini and Keum, 2018). EPA can also be metabolized to five series of leukotrienes (B_5_, C_5_, and D_6_), which has various anti-inflammatory effects (Saini and Keum, 2018; Sheikh et al., 2019). EPA also competes with arachidonic acid for cell membrane phospholipid synthesis, resulting in reduced production of thromboxane A_2_ and more production of TXA_3_, thereby inhibiting platelet activity (Sheikh et al., 2019). It is regarded as one of the important mechanisms of cardiovascular benefits of EPA.

Experts’ opinion 4: Existing studies suggest that both EPA and DHA in omega-3 fatty acids have the effect of improving blood lipid profile, and the cardiovascular protective mechanism of their non-lipid-lowering effects and the differences between EPA and DHA deserve further exploration.

## Recommendations for the use of omega-3 fatty acids in cardiovascular disease

According to the existing evidence, high-purity and high-dose EPA can significantly reduce the risk of cardiovascular events. However, it is not clear whether the mixture of EPA and DHA can bring cardiovascular benefits. Based on the REDUCE-IT study, IPE has become the only omega-3 fatty acid approved by the FDA, Canada, and the European Union (EU) for cardiovascular risk reduction (CRR) in patients with CVD or diabetes with other ASCVD risk factors (Barry and Dixon, 2021). Although EPA + DHA was approved for CRR in the EU, after review, EMAconfirmed that EPA + DHA 1 g/d is not effective in preventing future cardiac and vascular problems in patients with heart disease, and there is no favorable benefit-risk ratio in the secondary prevention of myocardial infarction. Hence, in 2019, approval for EPA + DHA for the reduction of CRR was revoked (European Medicines Agency, 2019). Therefore, in global guidelines and consensus, EPA + DHA mixed preparations are only indicated to reduce severe HTG, but not CRR. Based on the recommendations of the above guidelines, high-dose IPE can bring cardiovascular benefits to patients with elevated TG at high risk of ASCVD and other cardiovascular risk factors (such as diabetes mellitus). Therefore, highly purified EPA (IPE) is mainly suitable for primary and secondary prevention of ASCVD in patients with high cardiovascular risk. In addition, the 2022 American Diabetes Association (ADA) Standards of Medical Care in Diabetes emphasize that there is a lack of data on other omega-3 fatty acids, and the REDUCE-IT study results should not be extrapolated to other products. The Chinese and global expert consensus also recommend that high-dose IPE be used in high TG patients with high ASCVD risk and above (Chinese Society of Cardiology of Chinese Medical Association et al., 2020; Mach et al., 2020; Hypertensive Group of Chinese Society of Cardiology of Chinese Medical Association and Editorial Board of Chinese Journal of Cardiology, 2021; Kleindorfer et al., 2021; National Health Commissim Capacity Buiding and Continuing Education Center et al., 2021). The 2020 Chinese Expert Consensus on Secondary Prevention after Coronary Artery Bypass Surgery also recommends it for patients undergoing revascularization, and specifically States that EPA, rather than EPA + DHA, can further reduce cardiovascular events (Working Group of the Chinese Expert Consensus on Secondary Prevention after Coronary Artery Bypass Surgery et al., 2021; American Diabetes Association Professional Practice Committee, 2022). The Chinese and global Experts’ consensus also recommend that high-dose IPE be used in high TG patients with high ASCVD risk and above (Chinese Society of Cardiology of Chinese Medical Association et al., 2020; Mach et al., 2020; Hypertensive Group of Chinese Society of Cardiology of Chinese Medical Association and Editorial Board of Chinese Journal of Cardiology, 2021; Kleindorfer et al., 2021; National Health Commissim Capacity Buiding and Continuing Education Center et al., 2021). The 2020 Chinese Experts’ Consensus on Secondary Prevention after Coronary Artery Bypass Surgery also recommends it for patients undergoing revascularization, and specifically States that EPA, rather than EPA + DHA, can further reduce cardiovascular events (Working Group of the Chinese Experts' Consensus on Secondary Prevention after Coronary Artery Bypass Surgery et al., 2021).

Experts’ opinion 5: For patients at high/very high risk of ASCVD, if TG is still greater than 1.5 mmol/L (135 mg/dl), high-dose IPE (4 g/d) is recommended in adjunct with strict lifestyle intervention and statin therapy, to further reduce cardiovascular risk.

Based on the limited evidence available, omega-3 fatty acids mildly reduce the risk of hospitalization and all-cause mortality in patients with heart failure, but have inconsistent benefits in patients with arrhythmias, may be beneficial in patients with cardiomyopathy, and have a mild blood pressure lowering effect in hypertensive patients. Although the 2017 AHA Science Advisory on Omega-3 Polyunsaturated Fatty Acid (Fish Oil) Supplementation and the Prevention of Clinical Cardiovascular Disease recommends that it is reasonable to use omega-3 fatty acids for secondary prevention in patients with heart failure based on the GISSI-HF study (IIa, B) (Siscovick et al., 2017), and the latest 2022 AHA/ACC/HFSA Guideline for the Management of Heart Failure also recommends omega-3 fatty acids for patients with heart failure (NYHA Class II-IV) to reduce the risk of cardiovascular hospitalization and death (IIb, B). This is the first time that omega-3 fatty acids have been recommended from the perspective of heart failure treatment (Heidenreich et al., 2022). However, the evidence of omega-3 fatty acids is insufficient and shows only a modest benefit in arrhythmia, cardiomyopathy, hypertension, sudden cardiac death, etc, which is not enough to support the recommendation of relevant guidelines. Therefore, the role of omega-3 fatty acids in these diseases needs further observation. This Experts’ consensus summarizes the recently published guidelines and/or consensus in China and worldwide, and the basic information on the recommendation of omega-3 fatty acids for CVD is shown in Table 3.

**TABLE 3:** Recommendation of Omega-3 fatty acids for the treatment of cardiovascular diseases in major guidelines or consensus in China and worldwide.

Guidelines or consensus	Cardiovascular disease	Content
2022 American Diabetes Association (ADA) Standards of Medical Care in Diabetes American Diabetes Association Professional Practice Committee (2022)	ASCVD	In patients with atherosclerotic cardiovascular disease or other cardiovascular risk factors on a statin with controlled LDL cholesterol but elevated triglycerides 1.5–5.6 mmol/L (135–499 mg/dl), the addition of icosapent ethyl can be considered to reduce cardiovascular risk (A)
2021 American Heart Association (AHA)/American Stroke Association (ASA) Guideline for the Prevention of Stroke in Patients With Stroke and TIA (Kleindorfer et al., 2021)	ASCVD	In patients with ischemic stroke or TIA, with fasting triglycerides 1.5–5.6 mmol/L (135–499 mg/dl) and LDL-C of 1–2.6 mmol/L (41–100 mg/dl), on moderate- or high-intensity statin therapy, with HbA_1C_<10%, and with no history of pancreatitis, AF, or severe heart failure, treatment with IPE 2 g twice a day is reasonable to reduce risk of recurrent stroke. (Ⅱa, B)
2020 Chinese Guideline on the Primary Prevention of Cardiovascular Diseases Chinese Society of Cardiology of Chinese Medical Association et al. (2020)	ASCVD	High-dose IPE (2 g, twice a day) should be considered to further reduce the risk of ASCVD in patients at high risk of ASCVD after intermediate statin therapy if TG > 2.3 mmol/L (200 mg/dl)) (Ⅱa, B)
2019 European Society of Cardiology (ESC)/EAS Guidelines for the Management of Dyslipidaemias Mach et al. (2020)	ASCVD	In high-risk (or above) patients with TG levels between 1.5 and 5.6 mmol/L (135–499 mg/dl) despite statin treatment, Omega-3 polyunsaturated fatty acids (IPE 2 × 2g/day) should be considered in combination with statins (IIa, B)
2022 American Heart Association (AHA)/American College of Cardiology (ACC)/Heart Failure Society of America (HFSA) Guideline for the Management of Heart Failure Heidenreich et al. (2022)	Heart failure	In patients with heart failure class II to IV symptoms, omega-3 fatty acid may be reasonable to use as adjunctive therapy to reduce mortality and cardiovascular hospitalizations (IIb, B)
2021 ACC Experts’ Consensus Decision Pathway on the management of ASCVD risk Reduction in Patients with Persistent HTG Virani et al. (2021)	ASCVD	At the time of this publication, the only triglyceride risk-based nonstatin therapy approved for reduction in ASCVD risk by the U.S. Food and Drug Administration is IPE.
2021 Experts’ Consensus on the Management of Diabetic Patients with Cardiovascular Diseases National Health Commissim Capacity Buiding and Continuing Education Center et al. (2021)	ASCVD	Patients at high/very high risk of cardiovascular disease, on the basis of strict lifestyle intervention and statin therapy, tend to use high-dose IPE (2 g, twice a day) to further reduce the risk of cardiovascular disease if TG > 2.3 mmol/L (200 mg/dl)
2021 Experts’ Consensus on the Comprehensive Management of Blood Pressure and Dyslipidemia in Chinese Hypertensive Patients Hypertensive Group of Chinese Society of Cardiology of Chinese Medical Association and Editorial Board of Chinese Journal of Cardiology (2021)	ASCVD	High-dose IPE (2 g, twice a day) can reduce the level of TG and the incidence of cardiovascular events to a certain extent
2020 Chinese Experts’ Consensus on Secondary Prevention after Coronary Artery Bypass Surgery Working Group of the Chinese Experts' Consensus on Secondary Prevention after Coronary Artery Bypass Surgery et al. (2021)	ASCVD	For patients with HTG, highly purified EPA supplementation may be considered for secondary prevention to further reduce cardiovascular events, rather than omega-3 polyunsaturated fatty acids mixed with DHA
2017 Omega-3 Polyunsaturated Fatty Acid (Fish Oil) Supplementation and the Prevention of Clinical Cardiovascular Disease A Science Advisory From the American Heart Association (AHA) Siscovick et al. (2017)	Heart failure	It is reasonable to recommend omega-3 fatty acids for secondary prevention in patients with heart failure based on the GISSI-HF study (IIa, B)

Note: ASCVD, atherosclerotic cardiovascular disease; LDL-C, low density lipoprotein cholesterol; TG, triglyceride; IPE, icosapent ethyl; EPA, eicosapentaenoic acid; TIA, transient ischemic attack; HbA1C, glycosylated hemoglobin; HTG, hypertriglyceridemia; DHA, docosahexaenoic acid.

## Recommendations for omega-3 fatty acids in hypertriglyceridemia treatment

There is no doubt that omega-3 fatty acids can significantly reduce blood TG levels, which in turn affects the blood content of TRL-C. In a study published in 1997, 42 patients with TG levels ranging from 5.6 to 22.6 mmol/L (500–2000 mg/dl) were treated with omega-3 fatty acids (EPA + DHA) 4 g/d or placebo for 4 months. Omega-3 fatty acids reduced TG by 45% (*p* < 0.01), whereas placebo had no effect (Harris et al., 1997). The MARINE study, published in 2011, included 229 patients with TG ≥ 5.6 mmol/L (500 mg/dl) but <22.6 mmol/L (2000 mg/DL) randomized to IPE 4 g/d, 2 g/d, or placebo for 3 months and showed that, compared with placebo, IPE 4 g/d and 2 g/d reduced TG levels by 33.1% and 19.7%, respectively (both *p* < 0.01) (Bays et al., 2011). Based on these studies, the FDA approved EPA + DHA formulation and IPE in 2004 and 2012, respectively, as indications for lowering TG levels in adult patients with severe HTG [TG ≥ 5.6 mmol/L (500 mg/dl)] (Benes et al., 2018). At the same time, omega-3 fatty acids are also recommended by Chinese and global guidelines and/or consensus. It is recommended in Chinese and global guidelines that omega-3 fatty acids (IPE or EPA + DHA) should be added to severe HTG patients with TG ≥ 5.6 mmol/L (500 mg/dl) or even 11.3 mmol/L (1000 mg/dl) to reduce TG levels after excluding other causes and being treated with statins and other drugs (The Writing Committee of the Report on Cardiovascular Health and Diseases in China, 2022; Virani et al., 2021; Skulas-Ray et al., 2019; American Diabetes Association, 2019; Précoma et al., 2019; Grundy et al., 2019; Task Force on Chinese Guidelines for the Prevention of Cardiovascular Diseases (2017) and Editorial Board of Chinese Journal of Cardiology, 2018; Handelsman et al., 2020). Because severe HTG is one of the recognized causes of acute pancreatitis, some guidelines also recommend the use of omega-3 fatty acids in patients with severe HTG to prevent acute pancreatitis, but its impact on the acute phase of pancreatitis is not clear (Rawla et al., 2018). It is noteworthy that the 2019 AHA Science Advisory states that omega-3 fatty acid preparations containing DHA increase LDL-C levels (Skulas-Ray et al., 2019).

The ANCHOR study published in 2012 included 702 patients with HTG [2.3 mmol/L (200 mg/dl) ≤ TG < 5.6 mmol/L (500 mg/dl)] after statin treatment, who were randomized to IPE 4 g/d, 2 g/d, or placebo for 3 months. The results showed that IPE 4 g/d and 2 g/d reduced TG levels by 21.5% and 10.1%, respectively, compared with placebo (both *p* < 0.01) (Ballantyne et al., 2012). It was observed that the reduction of TG in omega-3 fatty acid treatment was significantly greater in patients with severe HTG patients, than that in HTG patients. HTG patients have an elevated risk of ASCVD, and the use of omega-3 fatty acids should be targeted at the improvement of cardiovascular risk, and be used rationally according to the recommendations of ASCVD prevention and treatment.

Experts’ opinion 6: Both EPA and (or) DHA preparations of omega-3 fatty acids have a positive effect on reducing TG and TRL-C in a dose-dependent manner.

## Adverse effects of treatment with 8 omega-3 fatty acids

Omega-3 fatty acids generally have a good safety and tolerability profile and have shown relatively few and mild adverse reactions in clinical studies, with less than 5% of patients discontinuing treatment due to adverse reactions. The most common adverse reactions of omega-3 fatty acids are mild gastrointestinal adverse reactions (fishy odor, belching, diarrhea, and nausea), but they can be reduced if taken with meals and can improve the absorption of omega-3 fatty acids (Skulas-Ray et al., 2019; Mach et al., 2020). It should be noted that omega-3 fatty acids may increase the risk of AF. Although previous randomized controlled studies have suggested that omega-3 fatty acids have no effect on the risk of recurrent AF or stroke in patients with AF (Mozaffarian et al., 2012; Sandesara et al., 2012; Macchia et al., 2013). However, studies assessing cardiovascular outcomes, such as REDUCE-IT, have found an increased risk of AF (Bhatt et al., 2019). 2017 AHA Science Advisory on Omega-3 Polyunsaturated Fatty Acid (Fish Oil) Supplementation and the Prevention of Clinical Cardiovascular Disease does not recommend omega-3 fatty acids for patients with AF (III, A) (Siscovick et al., 2017). Therefore, Experts need to evaluate the use of omega-3 fatty acids in patients with AF in clinical diagnosis and treatment. Omega-3 fatty acids also have antiplatelet effects and a slight increase in total bleeding events was observed in the JELIS study (1.1% vs. 0.6%, *p* = 0.0006). A trend toward increased bleeding risk was also observed in the REDUCE-IT study (2.7% vs. 2.1%, *p* = 0.06), but there was no increase in hemorrhagic stroke or fatal bleeding in either study. Therefore, the risk of bleeding should be monitored regularly when omega-3 fatty acids are used with anticoagulants or antiplatelet drugs (Skulas-Ray et al., 2019).

## Omega-3 fatty acids clinical application, future outlook

With regard to the effects of omega-3 fatty acids on cardiovascular events, only EPA has been found to confer cardiovascular benefits, including a reduction in TG levels and its possible pleiotropic effects. The benefit is not only related to the dose of EPA, but also to the level of serum EPA in the patients. Subgroup and/or *post hoc* analyses have found that omega-3 fatty acids are beneficial only in people with low blood EPA levels, but there have been no head-to-head studies comparing EPA with EPA + DHA, and no prescription formulations of DHA alone have been approved for marketing. Therefore, the effects of DHA and EPA on cardiovascular system and cardiovascular events need to be further studied to identify the population with maximum benefit, optimal composition and dosage of omega-3 fatty acids.

At the same time, due to the high degree of clinical heterogeneity in the design of previous studies (including the composition, source and dosage of omega-3 fatty acids, the characteristics of placebo and risk population selected in the study) and the final results, there is still disagreement in dealing with these heterogeneity and inefficiency in some studies (Siscovick et al., 2017; Barry and Dixon, 2021). Moreover, with regard to the possible negative effects of mineral oil as a control group on blood biomarkers in the REDUCE-IT study, it is expected that the design of future studies should be standardized and unified as far as possible.

In addition, although omega-3 fatty acids are generally well tolerated, safety information is mainly derived from short-term randomized clinical trials, and the incidence of gastrointestinal side effects may vary from product to product, requiring long-term monitoring studies.

Finally, the benefits of IPE in people with tightly controlled LDL-C levels or lower and the value of omega-3 fatty acids in other CVD, including heart failure, cardiomyopathy, and atrial fibrillation, need to be further clarified (Skulas-Ray et al., 2019; Barry and Dixon, 2021).

## Glossary

ACC: American college of cardiology
ACCORD study: action to control cardiovascular risk in diabetes
ADA: American diabetes association
AF: atrial fibrillation
AHA: the American heart association
ASCEND: A study of cardiovascular events in diabetes
ASCVD: AtheroSclerotic CardioVascular disease
CHERRY Study: combination therapy of eicosapentaenoic acid and pitavastatin for coronary plaque regression evaluated by integrated backscatter intravascular ultrasonography
CM: chyloMicrons
COX-2: cyclooxygenase-2
CRR: cardiovascular risk reduction
CVD: cardiovascular disease
DART: diet and reinfarction trial
DHA: docosaHexaenoic acid
EAS: european atherosclerosis society
EMA: European medicines agency
EPA: eicosaPentaenoic acid
EU: the European union
EVAPORATE study: effect of vascepa on the progression of coronary atherosclerosis in patients with elevated triglycerides (200–499 mg/dl) on statin therapy
GISSI: grupo italiano studiosopravvivenza infartomiocardico
GISSI-HF: grupo italiano studiosoPravvivenza infartomiocardico–heart failure
HDL: high-density lipoprotein
HFSA: heart failure society of america
Hs-CRP: High-sensitivity C-Reactive Protein
HTG: High Triglycerides
ICD: implantable cardioverter defribrillator
IL-6: InterLeukin-6 (IL-6),
IL-1β: InterLeukin-1β
ILDL: intermediate low density lipoproteins
IPE: IcosaPent Ethyl
JELIS: Japan EPA lipid intervention study
LDL: low density lipoprotein
LDL-C: low density lipoprotein cholesterol
Lp-PLA2: lipoprotein-associated phosphoLipase A2
MARINE study: eicosapentaenoic acid ethyl ester (AMR101) therapy in patients with very high triglyceride levels ( from the multicenter, plAcebo controlled randomized double blINd, 12 weeks study with an open label extension
MERLIN-TIMI 36 study: metabolic efficiency with ranolazine for less ischemia in non-st elevation acute coronary syndromes
MESA cohort study: multi ethnic study of atherosclerosis
NYHA–classification: New York heart association classification
ONEMI: omega-3 fatty acids in elderly with myocardial infarction
Ox-LDL-C: Oxidized LDL-C
PCI: percutaneous coronary intervention
PCSK9: proprotein convertase subtilisin/kexin type 9
**PGI**_3_: ProstaGlandin I_3_
REDUCE-IT: reduction of cardiovascular events with icosapent ethyl-intervention trial
RR: relative risk
SOFA study: study on omega-3 fatty acids and ventricular arrhythmia
STRENGTH: Study To assess statin Residual risk with EpaNova in hiGh cardiovascular risk paTiens with Hypertriglyceridemia
TG: triglyceride
TRL: triglyceride-rich lipoprotein
TRL-C: triglyceride-rich lipoprotein cholesterol
**TXA**_3_: Thromboxane A_3_
US-FDA: united states food and drug administration
VITAL-HF study: virtual care to improve heart failure outcomes
VLDL: very low-density lipoproteins
VTE: ventricularttachyarrhythmia episodes
5-LOX: 5LlipOXygenase
